# Supplementary material for: Are carbon emissions trading and green financial instruments synergistic? -Comprehensive quantitative research based on content analysis
Source: PLoS One. 2024 Mar 7;19(3):e0298601. doi: 10.1371/journal.pone.0298601 (PMC10919601; doi:10.1371/journal.pone.0298601)
Supplement: S1 Data — (ZIP) [file pone.0298601.s001.zip › Data/地级市Z_F工作报告.zip!/╣π╢1⁄2/╒╪╟∞╩╨/└┤╫╘╣1⁄2╓┌║┼í╛┬φ┐╦╩2╛▌═°í┐_2009.docx]

各位代表：

我代表肇庆市人民政府向大会作政府工作报告，请予审议，并请政协委员和其他列席人员提出意见。

2008年工作回顾

去年，在市委的正确领导和市人大及其常委会、市政协的监督支持下，市政府坚持以科学发展观统领经济社会发展全局，认真贯彻落实省委十届二次、三次全会和中共中央政治局委员、省委书记汪洋同志视察肇庆重要讲话精神，按照市委十届四次、五次全会工作部署，团结带领全市人民解放思想，开拓创新，扎实工作，积极应对国际金融危机带来的冲击，着力破解经济运行中的困难和问题，采取有力措施促发展、保稳定，全市经济社会保持较快发展态势，全面完成或超额完成了市十一届人大三次会议确定的目标任务，大部分经济指标增速超过全省平均水平，部分居全省前列。完成肇庆市生产总值715.85亿元，比上年增长14.2%，比全省平均增幅高4.1个百分点。人均生产总值增长12.7%，比全省平均增幅高4个百分点。一、二、三产业增加值分别增长5.1%、24.1%和11.4%，三大产业比重为22.7：36.7：40.6。

—— 工业经济快速发展。实现工业总产值突破千亿大关，达1061.96亿元。规模以上工业增加值226.43亿元，比上年增长30.4%，增幅排名全省第三；预计实现工业税金增长30%。

—— “双转移”抢占先机。中山（肇庆大旺）产业转移工业园成功竞得首批省级示范性产业转移园，获得省5亿元专项扶持资金；全市新增珠三角转移工业项目185宗，计划总投资217.92亿元。

—— 招商引资态势良好。新批外商投资项目189宗，合同投资总额17.91亿美元；实际吸收外资8.58亿美元，比上年增长10.8%，总量继续居全省东西两翼和山区市首位。

—— 重大项目加快推进。有12个项目列入“广东新十项工程”；列入市重点办跟踪协调的51个重点项目累计完成投资76.6亿元，完成年度计划的114.3%。一批生产性项目和公路、水路、水利建设项目顺利推进。

—— 旅游产业持续畅旺。主要景区（点）接待游客1358.12万人次，旅游收入65.8亿元，分别比上年增长17.9%和19%。

—— 县域经济活力增强。县域规模以上工业增加值127.33亿元，占全市比重56%；地方财政一般预算收入24.85亿元，比上年增长39.9%。高要、四会地方财政一般预算收入均实现两年翻番，财政综合增长率分别居全省67个县（市）第一、第二位，封开从末位跃升至第八位，广宁、怀集排名分别提升7位和3位。

—— 生态环保成效明显。关停落后产能企业48家，完成省下达化学需氧量和二氧化硫各削减1000吨的污染减排任务；规模以上工业增加值能耗下降10％，万元生产总值能耗下降3.5%。

—— 财税收入较快增长。地方财政一般预算收入43.56亿元，比上年增长33.03%，增幅排名全省第二；国税、地税收入分别增长19.4%和35.7%。

—— 人民生活不断改善。城镇居民人均可支配收入13642元，比上年增长11.1%；农村居民人均纯收入5872元，增长9.1%；年末城乡居民储蓄存款余额比年初增长17.5%；居民消费价格总指数上涨5.3%，低于全国、全省平均水平。

—— 民生工程建设加快。全面实现城乡免费义务教育，新型农村合作医疗基本覆盖农村常住人口，实施职业技能等各类培训57006人，落实保障性住房1552套，启动农村泥砖住房改造试点，农村公路建设加快，新创建省卫生村618个、生态文明村504个。

在经济保持较快增长的同时，我市社会各项事业全面发展，社会大局和谐稳定，人民群众安居乐业。一年来，市政府和各部门明确目标，强化责任，狠抓落实，全力推进各项工作。

一、坚持以工业为主导，全力加快工业经济发展。抓好项目引进和企业增资扩产，积极帮助企业化解困难，促进企业做大做强，新投产规模以上工业企业36家。优化产业结构，打造支柱产业，六大支柱产业实现产值占规模以上工业总产值比重达46.9%。产业集群培育有新突破，与省经贸委签订了“省市共建汽车发动机零部件先进制造业基地”协议，将获得扶持资金9000万元；高要获得“中国新型建材产业基地”称号，金利五金产业集群成为我市首个省级产业集群升级示范区；鼎湖山泉增资1亿元的三期生产基地建成，初步形成饮用水产业集群。有3家企业进入上市辅导期。企业技改和品牌建设力度加大，有63个技术改造、技术创新、节能、中小企业发展等项目列入国家和省专项发展计划，获得第一批扶持资金3356万元；完成工业技术改造投资23.18亿元，比上年增长60.9%；新增工业类省名牌产品10个、省著名商标6件。

二、积极实施“双转移”战略，大力抓好外向型经济。肇庆高新区发挥省首批示范性产业转移园并获得省5亿元扶持资金的效应，与金融机构签订了96亿元融资意向，加强园区基础设施和配套设施建设，引进项目36宗，投资总额12.6亿美元，实际吸收外资3.1亿美元，继续在全省69个省级开发区中名列第一；实现规模以上工业增加值39.59亿元，地方财政一般预算收入3.5亿元，分别比上年增长58%和66.2%。顺德龙江（德庆）产业转移工业园加快“一园三区”规划建设，已落户企业50家，总投资56.3亿元。中山大涌（怀集）产业转移工业园入园项目32个，总投资20.2亿元。广东肇庆工业园迁址鼎湖正式启动。把招商引资作为促进经济发展的重要举措，创新招商方式和服务机制，先后组织参加赴香港、日韩、东盟、中东、美国、欧洲等国家和地区开展招商推介活动，到中山、东莞等地举办经贸洽谈活动，收到明显成效。“肇庆—东莞实施‘双转移’战略经贸合作交流会”签约项目40宗，合同投资总额68.19亿元；2008“肇庆金秋”经贸洽谈会签约、动工、投产项目共318宗，投资总额472亿元；在省第五届山洽会上，我市签约项目47宗，合同总额142亿元。引导企业调整出口经营策略，协调做好退税、通关等服务，口岸货物进出口总值37.8亿美元，比上年增长14.4%；外贸出口总额24.33亿美元，增长12%。统筹抓好农村劳动力技能培训和就业引导，实现转移就业5.05万人。

三、高度重视农业农村经济，着力提高县域经济发展水平。积极推进农村工业化、城镇化和农业现代化，促进农村经济发展、农民增收。落实各项支农惠农政策，各项支农资金8.98亿元，列入省级以上农业建设项目56个。整治基本农田8.28万亩。抓好救灾复产和灾害防治，农业生产稳定发展，实现农业总产值255.02亿元，比上年增长5.2%。农业产业化、规模化、基地化水平提高，西江北岸优质水果产业带项目和怀集梁村平原优质粮食基地项目获中央财政连续三年、每年4000万元专项补助及省财政配套资金支持。粮食、蔬菜、畜牧等传统农产品实现大灾之年不减产，预计粮食总产量111.5万吨，连续7年完成省粮食工作考评任务，柑桔种植面积扩大至93万亩，产量增长25%。农民专业合作组织发展到287家，已有农业龙头企业109家，带动19.61万农户户均增收2088元。端州近半村委会年集体收入超千万元。积极发展现代林业，四会集体林权制度改革试点基本完成并逐步全面铺开。高要蚬岗镇南村成为省农村土地流转改革试点。县域工业壮大发展，高要投资2亿元以上的北新建材、光丰钢业、新明珠陶瓷项目超额完成投资计划。四会规模以上工业企业突破200家，落户亚洲金属资源再生工业基地企业58家，其中建成投产17家。怀集铁矿开采加工业等资源型产业继续发展壮大，纳税超千万企业达10家。德庆大亚木业等一批项目建成投产。广宁华南再生资源产业基地正式动工建设。封开华润水泥、威利邦木业等一批大项目顺利推进。县域第三产业发展加快，城镇建设水平提高，旅游接待能力增强，商贸流通日益兴旺。

四、扎实抓好城市经济和港口物流经济，促进第三产业稳步发展。加强城市规划建设管理，完成编制《肇庆市城市总体规划(2007—2020)》和《城东新区控制性详细规划》、《鼎湖发展概念性规划》等多项专项规划，肇庆城东新区基础设施项目二期工程、城区管道天然气项目顺利推进，端州七、八路扩建和星湖环境综合治污等工程全面完工，龟顶新城项目规划建设抓紧推进，建成肇庆城区应急供水系统和北岭二区市政道路等一批市政工程。加强旅游宣传推介，拓展省外市场，提升旅游品牌形象，“南国旅游休闲之都—肇庆”被评为“中国旅游休闲十佳品牌”，“肇庆千里旅游画廊”成为“中国十大生态休闲基地”。稳定发展房地产业，完成房地产投资54.32亿元，比上年增长15.1%；商品房销售面积和销售额分别达149.87万平方米和43.96亿元。金融部门积极支持地方经济发展，举办“政银合作交流会”，建成“肇庆金融业统一征信平台”，年末人民币贷款余额比年初增长22.3%，农村信用社在全省率先兑付央行专项票据8.85亿元。港口物流经济发展加快，肇庆新港一期工程全面完成，高新区珠江物流园陆路运输设施基本建成，完成港口货物吞吐量1343.64万吨；集装箱吞吐量36.42万个标箱，比上年增长39.2%。商贸业持续兴旺，端州星湖国际广场、高要凤凰酒店等一批大型商贸企业相继建成营业，社会消费品零售总额233.81亿元，比上年增长18.8%。

五、继续谋划建设重大项目，推动基础设施建设上新水平。抓好重大项目前期及促建工作。列入省重点项目的华润水泥项目完成投资5.11亿元，亚洲铝业二期工程建成试产，碧桂园现代家居、科威半导体和福田化工等一批投资规模大的工业项目建设进度加快。二广高速公路三水至怀集段完成投资20.6亿元，累计完成投资52.7亿元；珠外环高速公路江门至肇庆段、广肇高速公路二期和西江航道肇庆至封开界首段整治工程已动工建设。肇庆核电厂在省内率先正式进入项目可研阶段，肇庆电厂项目正式上报国家发改委。途经我市的南广铁路、贵广铁路项目前期工作全面开展。景丰联围加固工程进入全面施工阶段。全社会固定资产投资完成348.85亿元，比上年增长18.3%。

六、努力创新体制机制，增强发展活力。完成了第三轮（第二批）行政审批制度改革。政府投资项目代建制顺利推进，企业投资项目核准制和备案制基本建立。完善公共财政管理体制，开展了市本级财务核算信息集中监管和单位公务卡结算试点改革。大力推进国有企业改革，引入战略投资者重组风华集团，基本完成市属国有经济阶段性整合和中小企业改革工作。出台扶持中小企业发展的政策，加强信用担保体系建设，现有12家担保公司为160家中小企业担保贷款4.8亿元。取得上级支持，从去年6月1日起，我市平均电价每千瓦时降低6.38分，其中大工业电价、非普工业电价和商业电价分别比珠三角核心区同类电价低7分、6.6分和3分，每年可减轻企业负担3亿多元。

七、加大节能减排力度，重视保护生态环境。建立完善节能监督、统计、考核三个体系，全面完成对县级政府和50家重点耗能企业节能目标责任制考核。认真实施环保规划，严把环保审批关，加大环保执法力度，重点污染源在线监控系统建成运行，完成了40家限期治理企业整治，重点工业企业废水排放达标率达96%。8座污水处理厂动工建设。四会独水河综合整治加快推进。污染源普查完成阶段性任务。累计有11家企业获省“清洁生产企业”称号。肇庆城区空气环境质量优于国家大气环境质量二级标准。创建绿色学校、生态示范村（镇）深入开展。积极建设林业重点生态工程，德庆成为省“林业生态县”，怀集创建工作通过省验收。加强土地开发利用和集约节约用地，开发补充耕地6.5万亩，开发量全省第一；完成省下达年度耕地保有量和年度土地利用执行任务，实际新增建设用地规模增加，顺利通过省第三次卫片执法检查验收。

八、切实关注民生民利，努力改善群众生产生活条件。深入推进“五大民生工程”。实施下岗失业人员再就业培训3879人、农村青年技能培训34107人、农民工技能提升培训15163人、退役士兵培训388人，新增就业人数42251人，年末城镇登记失业率2.58％。全面实现城乡免费义务教育，减轻群众负担3.2亿元；投入1.3亿元改善薄弱学校办学条件。加快城市社区卫生服务机构建设，抓紧推进乡镇卫生院危房改造，新建成规范化村卫生站1015个。参加新型农村合作医疗农民达284.17万人，常住人口参合率达99.57%，市、县两级财政配套资金标准提高到每人每年25元。多形式、多渠道落实廉租房564套，通过发放租赁补贴解决119户困难家庭住房问题。完成通500人以上大自然村道路硬底化建设612公里，新开农村公路385公里，实现通等级公路行政村通客运班车。完成2000个户用型沼气池建设。基本解决9.13万人的饮水不安全问题。社会保险五大险种参保人数超额完成目标任务。城镇居民基本医疗保险参保登记人数完成省下达任务的100.93%，财政补助标准提高到每人每年90元。企业退休人员人均养老金比上年增长11.6%。端州区被确定为全国“农村养老保障工作重点联系县(区)”。积极帮助灾民重建家园，2500户灾民喜迁新居。发放低保资金5832万元，实现人均年纯收入低于1500元的贫困家庭应保尽保。全力支援汶川抗震救灾，共筹集和发动社会各界捐款1.533亿元，财政预算安排援川资金3274万元，开展了克枯乡对口重建工作。

九、大力发展社会各项事业，促进社会和谐稳定

积极推动科技进步。组织专业技术人员参加教育培训4.45万人次，引进本科学历和中级职称以上各类人才4677人。新认定省级高新技术企业16家、省级民营科技企业9家，建成工程技术研发中心4家、技术创新专业镇11个。高新技术产业产值136亿元，比上年增长20.7%。市科技中心建成启用。接受科普教育群众超过100万人次。成立了肇庆汽车零部件省部产学研创新联盟。成为省级光电产业基地和科技金融试点市。荣获“中国城市信息化创新奖”，“农村信息直通车工程”走在全国前列。

优先发展教育事业。义务教育“普九”巩固率居全省前列，有效缓解端州城区小学学位紧缺问题。高中阶段教育再上新水平，新增高中阶段教育学位1.58万个，毛入学率72.6%，超过全省平均水平。职业技术教育发展速度和规模居全省前列。师资队伍素质不断提高，组织教师培训4.5万人次，招聘、引进中小学骨干教师1500多名。创建省级教育强镇2个，国家示范性高中1所，省普通高中教学水平评估优秀学校3所。有6个县(市、区)被省政府授予“广东省教育收费规范县”称号。高等教育取得新成绩。乡镇中心幼儿园开办率居全省第一。三残儿童入学率达94.8%。

加快发展文化事业。加强文化设施建设，完成广播电视村村通工程和中央节目无线覆盖工程建设，市、县、镇、村四级文化设施网络基本建成，启动了“数字图书馆”建设。开展文化创作年活动，推出一批艺术精品，组织了系列大型文化活动。文物古迹和古村落保护工作得到加强，端砚文化特色产业集聚园、古宋城保护与开发利用项目列入“广东新十项工程”，端砚成为北京奥运会贵宾礼品，端砚文化村被文化部命名为“第三批国家文化产业示范基地”。

全面发展其他社会事业。加强公共卫生服务体系建设，医疗管理不断规范，疾病防控水平得到提高，中医药事业发展加快，累计创建省卫生村996个。产品质量和食品、药品安全专项整治工作取得成效。深入开展全民健身活动，组织和举办了系列重要体育赛事，竞技体育成绩突出，冼东妹蝉联奥运金牌，覃小俊获残奥金牌。“两委”换届选举顺利完成。完成了省下达的年度人口控制计划，人口出生率和自然增长率分别为11.47‰和6.22‰，分别比省下达计划低0.73和0.78个千分点。第二次经济普查进展顺利。国防动员工作不断加强，连续第五次被命名为全省双拥模范城。民族、宗教、人防、外事、侨务、港澳、妇女儿童、老龄、残疾人、档案、市志等工作取得新成绩。

确保社会大局和谐稳定。“五五”普法深入推进。开展重信重访问题专项治理和“干部大下访”活动，建立市、县、镇三级党政领导定期公开接访制度，有效化解矛盾纠纷。深入推进“平安肇庆”建设，108个乡镇（街道）全部建立了综治工作中心（群众工作站），创建平安和谐社区、平安和谐村扎实有效。评选表彰了十佳外来工和十佳外来工用工企业，构建和谐劳资关系取得成效。落实安全生产责任制，安全生产形势平稳。加强社会治安综合治理，严厉打击各类犯罪活动，通过了中央综治委检查组创建2005—2008年度全国社会治安综合治理优秀市检查验收。

十、不断加强政府自身建设，进一步提高公共服务水平。认真执行市人大及其常委会的决议，向市人大及其常委会提交各类专项工作报告20件，组织办理人大代表建议73件。向市政协通报工作情况，组织办理政协提案142件。改进政府公共服务，完善经济目标责任考核和机关建设作风测评，启动了科学发展绩效考核。依法按时处理行政复议申请35件。组织公务员参加各类培训2.92万人次。健全市行政服务中心网上审批系统，受理（办理）审批事项48008宗，办结率100%。市政府门户网站被授予“中国城市政府门户网站十大影响力品牌”。县级“行风热线”全面铺开。审计监督得到加强。政府应急处置能力提高。落实政府廉政工作责任制，加强了行政监察，严肃查处违法违纪行为。各级驻肇单位为我市经济社会发展作出了积极贡献。

各位代表，去年市政府工作的顺利完成，是在省委、省政府和市委的正确领导，以及市人大、市政协的监督支持下取得的，凝聚着全市上下、社会各界的智慧和汗水。在此，我代表市人民政府，向广大工人、农民、干部职工、知识分子、外来务工人员、驻肇单位、驻肇部队、武警官兵以及各界人士致以崇高的敬意！向关心和支持肇庆建设发展的外来投资者、港澳台同胞、海外侨胞和国际友人表示衷心的感谢！

在总结成绩的同时，我们也清醒看到经济社会发展中存在的困难和问题：经济总量偏少，人均水平不高，区域经济发展不平衡仍然突出；国际金融危机影响日益明显，不少企业生产经营困难，财政增收难度加大；缺乏具有带动和支撑能力的骨干企业，自主创新、市场开拓不足，抗击风险能力相对较弱；城市建设水平有待提高，部分重大项目和城区建设项目由于征地拆迁难度大、资金不足等原因受到制约，未能按计划进度完成；农业基础设施建设存在薄弱环节，农资价格高企，农产品价格下降，农民增收难度较大；社会保障水平还不高，不少民生热点难点问题有待解决；机关作风有待进一步改进，社会管理、公共服务和市场监管能力仍待加强，这些都需要采取切实有效的措施加以解决。

2009年工作安排

2009年是新中国成立60周年，也是我市实现“十一五”规划目标的关键年。做好今年工作，必须正确把握形势，科学谋划发展。当前，国际金融危机的影响日益加深。我市去年9月份以来工业增加值、固定资产投资、外贸出口、商品住宅销售等增幅明显回落。很多企业正常经营受到冲击，普遍存在订单减少、库存增加、出口下降、资金严重不足等问题，群众生产生活中的热点难点问题也增多。我们必须保持清醒的头脑，做好应对困难的准备，增强工作的紧迫感和责任感。同时，更要抢抓发展新机遇，坚定发展信心。去年年底，国家决定实施积极的财政政策和适度宽松的货币政策，把保持经济平稳较快发展作为今年经济工作的首要任务，出台一系列保增长、扩内需、调结构的政策措施，计划今明两年投资4万亿元。我省出台了16项促进经济平稳较快发展的政策和扶持中小企业平稳健康发展的意见，计划今年总投资1.3万亿元，省财政安排400多亿元，用于建设现代产业体系、实施“双转移”战略和扶持中小企业。这些政策措施为我市化解当前困难、实现新发展提供了机遇条件。国务院批准实施《珠江三角洲地区改革发展规划纲要》，不仅为珠三角地区科学发展、先行先试带来重大战略机遇，而且将肇庆纳入珠三角区域规划，为我市加强与珠三角核心区的产业对接、同城发展、优势互补、扩大合作提供了难得的机会。经过改革开放30年全市广大干部群众的共同努力，我市经济基础更加坚实，基础设施不断完善，综合实力和竞争力逐步增强。我市还拥有丰富的资源、优良的环境和相对广阔的发展空间等后发优势。我们一定能够经受住挑战和考验，实现经济平稳较快增长，加快构建和谐社会。

今年市政府工作的指导思想是：深入贯彻落实党的十七大、十七届三中全会、中央经济工作会议和省委十届四次全会精神，按照市委十届六次全会的部署，以科学发展观统领经济社会发展全局，以实施珠三角改革发展规划纲要为契机，继续解放思想，坚持改革开放，转变发展方式，破解发展难题，继续实施“五大战略”，加速发展“四大经济”，大力推进“五大民生工程”，全力保增长、扩内需、调结构，着力保持经济平稳较快发展。坚定不移推进和谐社会建设，全面发展社会各项事业，加快建设繁荣活力、文明法治、和谐安康、生态环保肇庆，为把我市建设成为未来广东发展的新增长极和能够代表广东科学发展成果的城市打下更坚实基础。

经济社会发展主要预期目标建议如下：肇庆市生产总值增长12%，人均生产总值增长10.6%，一、二、三产业增加值分别增长5%、17.2%、11%。工业增加值增长18.8%，全社会固定资产投资增长20%，地方财政一般预算收入增长13%，社会消费品零售总额增长16%，实际吸收外资和合同利用外资有所增长，外贸出口总额增幅达到全省平均水平，万元生产总值能耗下降3.33%，城镇登记失业率控制在3%以内，人口自然增长率控制在省下达指标以内，城镇居民人均可支配收入增长9%，农村居民人均纯收入增长7%，居民消费价格总指数涨幅控制在4%以内。

一、积极应对宏观经济形势变化，努力推动经济平稳较快发展

（一）认真实施珠三角改革发展规划纲要，确保宏观经济政策措施落实到位。切实加强珠三角改革发展规划纲要的学习研究，深刻领会精神实质，统一认识，转变观念，把握机遇，按照国家和省的部署，抓紧制定我市实施意见，组建强有力的领导机构和工作班子，加紧与省对口部门衔接，结合实际制定新的专项规划或修编原有规划，加快全面融入珠三角发展步伐，积极与珠三角核心区交通设施对接、产业配套对接、城市建设对接、经济要素对接，打造传统优势产业转型升级集聚基地、先进制造业基地、资源型工业基地，积极发展高新技术产业、现代服务业，建立现代产业体系。认真贯彻执行国家、省的宏观经济政策措施，促进提高自主创新能力，促进传统产业转型升级，促进建设现代产业体系，保持经济平稳较快增长。抓紧落实已报项目审批和再筛选一批优质项目上报；积极争取国家和省加大对我市重大基础设施、产业发展、民生工程、生态环境和社会事业等方面的投入；落实增值税转型、调高出口退税率、支持中小企业发展的措施，提高企业应对困难、参与市场竞争能力。抓好我市21项扶持企业发展措施的落实到位，适当调减部分收费标准，缓缴部分收费项目，市财政安排3000万元筹建市中小企业信用担保公司；积极向金融机构定期推荐优质企业，争取金融机构扩大信贷规模并提高贷款到位率；优先安排重点项目建设用地，确保企业正常用电，着力解决制约企业发展的瓶颈问题。

（二）坚定不移发展壮大工业经济，扩大和增强产业规模实力。把支持工业企业稳定发展作为当前工作重点，做好跟踪服务，确保工业经济平稳较快发展。加快产业结构调整和优化升级，推动产业集聚，发展先进制造业、高新技术企业和资源型工业，推动主导产业向产业链高端延伸，增强配套发展能力。办好省市共建汽车零部件基地、省级光电产业基地、肇庆金属新材料产业基地和高要金利五金省级产业集群升级示范区，打造一批在行业占据竞争优势的产业集群。抓好工业企业促建促产和技改创新，重点推进250个新建、技改和增资扩产项目建设，抓好亚洲金属资源再生工业基地二期、亚洲铝业二期、石井水泥二期、鼎丰30万吨纸浆、华南再生资源产业基地等重大工业项目，促进华润水泥加快投资建设进度，力争首期年产400万吨水泥的两条生产线建成投产，启动封开铜钼矿资源开发。加强产学研合作联盟和企业技术研发中心建设。推动企业创名牌、争上市，再创一批国家级、省级品牌和著名、驰名商标，争取高要天龙油墨、高新区科茂树脂、端州华锋铝箔等企业上市融资。促进企业加强管理，提高经济效益。

（三）积极发展现代农业，加快建设社会主义新农村。认真贯彻落实党的十七届三中全会和中央农村工作会议精神，把深化农村改革作为解决“三农”问题的突破口，努力实现农业增效、农民增收，推进新农村建设。创新农业发展体制机制，探索土地承包经营权流转模式，力争两年内基本完成集体林权制度改革。加强农业基础设施建设，抓好农业综合开发和标准农田示范区建设，完成8万亩基本农田整治。发展现代农业，扶持农业龙头企业、农民专业合作组织和农产品行业协会，加快农产品加工流通体系建设。抓好西江北岸优质水果产业带、怀集梁村平原优质粮食基地建设，形成国家级柑桔产业带。完善农业科技推广体系，推进农业标准化生产和农产品质量认证，实施优质水稻工程，抓好无病毒种苗繁育基地建设，提高禽畜、水产品的良种率，争创农业品牌。加强动植物疫病防控，确保农产品安全。发展现代林业，加快建设现代林业示范基地，抓紧做好南方珍贵树种良种选育与扩繁中心（基地）建设。加强中心镇规划建设，提高城镇化水平。深入开展创建省卫生村和生态文明村活动，力争新建省卫生村450个、生态文明村500个。

（四）大力实施“双转移”战略，提高内外源经济发展水平。坚持把招商引资作为加快发展的主引擎，积极承接产业转移，抓好工作目标的落实和考核。肇庆高新区要用好用活省示范性产业转移园扶持资金，放大配套融资，引导各类资本参与园区开发，促进基础设施和公共服务功能升级，增强产业承接能力，争取成为国家级高新区和中央企业落户广东的重要基地。按照省级产业转移园建设标准把德庆和怀集两个产业转移园打造成为区域品牌园区。依托肇庆新港高标准规划建设广东肇庆工业园，港园结合发展临港工业，继续提升端州三榕工业园等现有县（市、区）工业园建设水平。以港澳台、日韩、欧美、中东等地为重点，组织好各项招商经贸活动，加强与广州等珠三角核心地区的产业对接，密切与中央和省属国有大型企业联系沟通，引进更多投资强度大、产业关联度高的项目。积极开展产业招商、园区招商和资源招商。加强对重大招商项目跟踪服务，促进在谈项目落实签约、已签约项目入资建设、在建项目投产达标。完善大通关协调联动机制，扶持有自主知识产权、自主品牌和高附加值的产品出口，推动加工贸易企业转型升级，努力扩大出口。落实扶持民营企业发展的政策措施，完善中小企业社会服务体系，提高民营企业应对风险困难能力，做大做强民营经济。积极组织劳务输出，加快发展劳务经济。

（五）继续谋划建设重大项目，着力扩大投资规模。加强珠三角改革发展规划纲要内容的细化研究和实施的具体工作，以加快交通基础设施建设为先导、以产业集聚发展为重点，积极谋划和推进重大项目建设。我市经过筛选和争取，已有22项初步列入了省今年重点建设项目计划，比去年增加11项，年度计划投资87.17亿元。包含初步列入省计划的重点建设项目，我市今年将组织建设投资项目和民生工程项目84项，年度计划投资209.93亿元。其中，交通项目15项，投资66.53亿元；能源项目3项(含电网建设改造工程)，投资18.31亿元；市政项目2项，投资11.4亿元；农林水利项目6项，投资7.8亿元；园区建设项目2项，投资11.66亿元；环保项目11项，投资9.39亿元；文化、旅游基础设施项目8项，投资4.68亿元；民生工程项目7项，投资15.37亿元；生产性及其他项目30项，投资64.79亿元。这些重点工程项目事关我市长远发展和民生民利，尤其是已上报和已开展项目可研等前期工作的肇庆核电、肇庆电厂和国电热电联产等一批重点能源项目必须全力以赴，务求新突破。

（六）加快发展城市经济和港口物流经济，提升第三产业发展水平。实施城市旅游发展规划，促进城市建设与旅游配套设施融合协调；创新旅游管理体制，推进星湖景区经营模式改革；丰富“千里旅游画廊”内涵，抓好端砚文化村、古村落等新景区景点的开发建设，充分利用推行国民旅游休闲计划和“旅游下乡”活动的契机，发展休闲度假旅游产品,加快星级酒店改造建设，打造“南国旅游休闲之都”。落实稳定发展房地产政策，加快商住配套设施建设，推动房地产业持续健康发展。加快发展现代服务业，推进特色购物和休闲娱乐区规划建设，扩大商贸、餐饮、娱乐网点，发展会展、电子商务、文化创意、信息服务等新兴产业。编制完成《肇庆港口总体规划》，协调推动铁路、公路、水路联运，加快肇庆高新区珠江物流园、肇庆粮食物流基地、肇庆新港二期工程和保税区建设，抓好高要港、马房港口岸扩建改造工程和封开港（华润）码头建设，发展壮大港口物流经济。

（七）加强产业辐射带动能力，促进区域经济协调发展。优化产业布局，发展壮大以肇庆高新区为核心的市直工业，扶持壮大风华高科、星湖科技、蓝带啤酒、亚洲铝业、本田金属等龙头骨干企业，发展电子信息、生物制药、食品饮料、金属加工、汽车配件等支柱产业，提升市直经济对全市的辐射带动能力。加快经济发展中心区与珠三角核心区的协调对接，推动资源共享、产业配套、交通一体、环保同治，发展先进制造业、加工制造业和高新技术产业，打造科学发展先导区域。加大山区资源集约开发力度，依托工业园区、产业集聚基地和专业镇，壮大产业规模实力；落实“家电下乡”等消费政策，推进“万村千乡”市场工程，扩大农村地区消费，繁荣发展商贸物流和特色旅游产业，努力使山区县跟上全市发展步伐。

（八）加强财税和金融工作，切实做到增收节支。拓展生产经营领域的财源和税基，支持财税部门完善征管手段，加强税收征管，强化非税收入管理，积极推动政府性资源市场化运作，增强政府可支配财力。加强财政支出管理，严格控制一般性行政经费支出，实行公务购车和用车经费、会议经费、公务接待费用、行政机关出国（境）费用、办公经费预算“五个零增长”。同时，严格控制由财政出资或行政机关主办的晚会、展览、庆典、论坛活动，争取这些活动比去年实现“四个减半”。加强政府性投资项目监管，落实政府性贷款“借用还”机制。推进政银企合作，落实中小企业融资担保扶持政策，支持信用担保公司拓展业务，争取开展小额贷款公司试点，构建多层次信贷服务体系。

二、努力优化发展环境，增强经济发展后劲

（一）加快基础设施项目建设，进一步增强投资吸引力。抓住国家、省加快基础设施建设的契机，加快公路、铁路、电力、航道、港口码头、水利等基础设施建设。力争二广高速公路三水至怀集段基本建成通车；珠三角城际轨道广州至肇庆段已确定今年8月动工，抓紧配合做好相关工作，力争3年完工；全面铺开珠外环高速公路江门至肇庆段、广肇高速公路二期、广梧高速公路封开连接线建设；开展国道321线小湘至马房段文明样板路升级改造；启动二广高速公路怀集至广西省界和至连州市界、肇庆城区至珠外环高速公路连接线、珠外环肇花高速公路等项目规划建设；协调推进西江航道肇庆至封开界首段整治工程，配合做好贵广和南广铁路肇庆段项目建设。协调推进能源电网设施改造升级，加快500千伏砚都变电站扩建等一批电网工程建设。加快城乡水利防灾减灾工程、景丰联围加固工程建设和河流小流域治理，完成中小型病险水库加固工程。

（二）加强城市规划建设管理，进一步提升城市品位。加快《肇庆市城市总体规划（2007—2020）》的报批实施,加强与珠三角核心区同城发展规划配套，强化中心区城市统一规划和控制管理，编制肇庆城区公共设施、旧城改造、公共交通、电力电网等专项规划。加快市政工程和公共交通建设，完成肇庆城东新区基础设施项目二期工程并启动三期工程，推进星湖大道、信安路改造升级，加快实施端州龟顶新城开发项目，推进鼎湖山新入口和坑口片区规划建设，促进公共服务向鼎湖延伸，推进城区一体化。抓好端州区“城中村”改造，新建东调洪湖公园、梅庵岗公园、龙顶岗公园,提高城区园林绿化水平，确保通过国家园林城市复检。全面铺开端州城区管道天然气工程，形成5万户供气能力。加强对临时建筑、违章建筑、占道经营和脏乱差的整治，提高城市管理水平。力争获得“中国人居环境范例奖”。

（三）强化环境保护和资源节约，进一步优化生态环境。推进污染减排，确保完成省下达的减排任务。落实节能降耗责任制，关闭淘汰落后产能，做好能源、原材料、水资源的节约使用，推行清洁生产，发展循环经济。把好项目准入关、审批关，引导重污染行业企业入园管理和集中治污。加快环保基础设施建设，确保市第三污水处理厂、高新区污水处理厂首期、四会污水处理厂二期和德庆、广宁、封开、怀集、鼎湖等县（区）污水处理厂年内建成，动工建设鼎湖、四会、高要中心镇污水处理厂。强化环保执法监管，以独水河流域整治为重点，加强河涌整治。做好国家环保模范城市复检和省文明城市复评迎检工作。加强森林资源、自然保护区及野生动植物的保护。基本完成市级土地利用总体规划大纲修编，全面编制县镇两级土地利用总体规划，推进基层国土所建设，完善农村宅基地管理，开展征地改革试点，加快第二次土地调查，探索建立节约集约用地分级考评制度，积极推进园地山坡地开发补充耕地工作，改造盘活“三旧”（旧城镇、旧村庄、旧厂房）用地，清理闲置土地，整合土地存量资源，大力查处违法违规用地，确保通过省第四次卫片执法检查。规范矿产资源管理，加快矿业权市场建设步伐。加强地质灾害防治和环境保护，推进矿山地质环境恢复治理。

（四）着力创新体制机制，进一步增强发展活力。开展第四轮行政审批改革。全面实行企业投资项目核准制和备案制。推进政府投资项目代建制。完善公共财政管理，推进国库集中支付、公务卡结算、财政支出绩效评价等财政监管制度改革。深化价格体制改革，规范医疗服务收费和药品定价，探索实施生活用水阶梯式计价办法，推进环保收费制度改革。加快整合全市公路收费站并实行年票制。推进市属国有经济阶段性整合，引入战略投资者重组市直重点企业，全面完成市属中小企业改革。

三、高度重视民生民利，着力改善群众生产生活环境

（一）继续推进“五大民生工程”，努力为群众办好事实事。推进职业技能培训工程,抓好下岗失业人员培训、农民工技能培训和创业培训。重点实施“一户一技能计划”，落实农村劳动力技能培训普惠制和农村贫困家庭子女免费接受职业技术教育政策,推动农村劳动力转移就业，力争成为全省统筹城乡就业示范市。推进教育均衡发展工程，完善免费义务教育经费保障机制和农村义务教育阶段学校校舍维修改造长效机制，统筹义务教育规范化学校建设，实施农村中小学装备工程和信息化工程，探索保障进城务工人员子女平等接受教育的有效办法。推进便民廉医工程，实现农村常住人口全部参加新型农村合作医疗，实际住院总补偿水平不低于住院总费用的40%；加快两市两区社区卫生服务中心和卫生服务站建设，推进乡镇卫生院改造，基本建成行政村一村一个规范化卫生站。推进全民安居工程，加快经济适用房和廉租房建设，落实端州城区700套保障性住房。完成农村泥砖住房改造试点并逐步推开。推进群众生产生活环境改造工程，加快城区道路和农贸市场的升级改造。加快通500人以上大自然村道路硬底化改造和农村危桥改造，完善农村客运网络，加强农村公路养护管理。解决农村30万人的饮水不安全问题。

（二）不断完善社会保障体系，增强社会保障能力。扩大社会保险征缴范围，完善养老保险市级统筹，配合做好企业职工基本养老保险省级统筹，提高企业退休人员养老保险待遇。扩大城乡一体化的医疗保险覆盖范围，探索实施“基本医疗保险普通门诊、市级、城乡三项统筹”模式。抓好端州、德庆新型农村养老保险试点。推进被征地农民、早期离开国有集体企业人员等群体的社会保障工作。提高城乡低保补助，在确保人均年纯收入低于1500元困难群众实现应保尽保基础上，争取把低于2000元的群众纳入低保范围。加强优抚安置、救助、救灾、慈善工作，加快敬老福星工程建设，争取五保户集中供养率达到30%以上，建设市级救灾应急庇护场所和市儿童福利院。

（三）高度关注热点难点问题，努力纾解民困。努力促进和扩大就业，落实积极就业政策，千方百计稳定现有就业岗位，培育新的就业增长点，鼓励自主创业，完善城乡就业援助制度。强化劳动监察执法，加快劳动保障违法行为网络监控工程建设，完善多方同步预防欠薪机制和企业劳动保障分类监控管理制度，健全破产倒闭企业劳动保障应急预案，协调处理劳资纠纷，构建和谐的劳资关系。加强法律援助，维护群众合法权益。帮助农村贫困家庭、城镇困难家庭、下岗人员、残疾人等困难群体解决基本生活问题。强化“打假”工作责任制，加强市场价格监测，规范行业收费管理。抓好端州城区交通整治，缓解交通阻塞问题。

（四）全力以赴，完成克枯乡对口扶持年度工作任务。认真落实援建汶川县克枯乡总体工作方案，足额及时安排财政援建资金，继续动员社会各界参与灾区重建。加快建设克枯乡小学、卫生院、自来水厂等第一批援建项目，确保克枯乡小学今年秋季建成使用。

四、全面发展社会各项事业，建设和谐安康肇庆

（一）努力推动科技进步。制定实施《引进和培养高层次人才实施办法》，优化人才工作政策环境，实施高校毕业生“三支一扶”计划，加强人才培养和引进，壮大人才队伍，优化人才结构。大力推进自主创新，落实企业自主创新激励机制，发挥市科技中心功能作用，提升肇庆高新区创业服务中心的研发转化能力，办好国家火炬计划肇庆新材料产业基地，推进省级科技金融试点市建设，完善科技服务体系。办好汽车零部件省部产学研创新联盟，实施一批汽配技术研发项目。拓展生物制药、金属加工等主导产业产学研合作平台。加快信息化建设，打造“数字肇庆”，推进科技信息直通车工作，依托中巴软件园和端州信息服务产业园发展软件、动漫等新兴产业项目。加快优质果品、水产星火技术产业带建设。加强科普、地震、专利和知识产权保护工作。

（二）加快建设教育强市。巩固和提高“普九”成果，确保各项指标达到或高于国家和省的要求，实现端州城区小学新生入学年龄降至6周岁。多渠道筹集9亿元资金新建扩建高中阶段学校，新增3万个高中阶段学位，力争今年提前完成普及高中阶段教育任务，高中阶段教育毛入学率达到85%以上。加快肇庆职教基地建设，建成市职业教育实训中心，力争创建国家级和省级重点职校各1所，争取成为我国南方重要职业技术教育基地。继续实施“强师振教”工程，依法落实中小学教师工资福利待遇“两相当”政策，切实解决代课教师历史遗留问题。支持高等院校调整优化专业设置，提高办学质量，推进肇庆医专鼎湖新校区建设。加快发展学前教育。重视发展特殊教育。

（三）促进文化繁荣发展。深化文化体制改革，推进文化投资体制多元化，抓好文化单位体制改革试点和乡镇综合文化站改革试点。加强文化产业发展规划，整合文化资源，发展文化产业，培育和引进一批文化策划包装中介机构，打造端砚文化、宋文化、岭南文化、龙母文化等特色文化品牌，积极培育艺术精品。建设市新图书馆、市粤剧演艺中心，改造市群众艺术馆，推进文化信息共享工程、农家书屋工程，完善公共文化服务体系。组织开展第四届肇庆读书节。继续抓好第三次全国文物普查工作，加强对非物质文化遗产的普查、保护和利用。

（四）大力发展卫生等社会各项事业。加强公共卫生服务体系建设，建成市疾控中心新大楼，加强疾控专业队伍建设，抓好人禽流感、艾滋病、狂犬病等疾病防治，积极实施“妇幼安康工程”。加强卫生信息网络建设，建成全市三级卫生监督网络。实施“基层卫生人才培养工程”。推进市第一人民医院迁址规划建设，争取把市中医院建设成国家重点中医院和省中医名院。完善人口计生工作新机制，扎实开展乡镇（街道）无政策外多孩出生和村（居委会）无政策外出生活动，加强人口计生基础建设，推进人口计生工作重心下移，提高流动人口计生服务水平，加快人口计生三类地区转化步伐，力争全市人口计生工作达到全省中上水平并向先进行列迈进。做好我市第六次全国人口普查准备工作。组织开展群众性体育健身活动，抓好第十三届省运会和第十一届全运会备战工作。加强国防动员和民兵预备役工作，完善国防应急动员机制，推进国防教育基地建设，建成人防应急指挥中心，做好拥军优属工作。落实民族和宗教政策。强化殡葬工作责任制。加强审计、外事、侨务、港澳、法制、统计、档案、市志、妇女儿童、残疾人等工作。

（五）切实保持社会稳定。完善应急机制和应急预案，提高应急处置能力。加强人民调解、司法调解与行政调解的衔接工作。推进平安县（市、区）、乡镇（街道）综治工作中心（群众工作站）和村（社区）综治工作站建设。认真排查和积极化解各类矛盾纠纷，强化信访维稳责任制，完善市、县、镇三级党政领导定期公开接访制度。推进社会治安综合治理，坚持打防结合，有效遏制和严厉打击各类违法犯罪活动。认真解决群众关心的治安热点问题，完善和推进社会治安视频监控系统建设。加强产品质量和食品、药品安全监管，推行食品卫生和公共场所卫生量化分级管理制度。落实安全生产监督责任制和企业安全生产主体责任，突出抓好道路交通、非煤矿山、尾矿库、危险化学品、渡口渡船安全生产和人员密集场所消防安全。

五、强化服务型政府建设，提高推动科学发展能力

（一）严格依法行政。贯彻落实国务院《全面推进依法行政实施纲要》和《加强市县政府依法行政的决定》。自觉接受人大及其常委会依法监督和市政协的民主监督，落实市人大及其常委会各项决议决定，执行向人大及其常委会报告工作和向人民政协通报情况制度，提高办理人大代表建议和政协委员提案的质量，听取民主党派、工商联、无党派人士和人民团体的意见建议。深入推进“五五”普法教育。落实行政执法责任制和过错责任追究制，推进相对集中行政处罚权和综合执法工作。推行政务、村务、厂务公开，健全基层和企事业单位民主管理制度，完善社会公示和听证制度，保障人民群众的知情权、参与权、表达权和监督权。

（二）强化队伍建设。认真组织政府系统开展科学发展观学习实践活动。完善科学发展观政绩考核评价制度。继续抓好公务员法及其配套法规的实施，创新公务员管理监督和激励约束机制。开展学习型机关创建活动，探索实施新录用公务员基层锻炼办法，提高公务员队伍综合素质，增强落实上级政策法规、支持基层和企业发展以及化解困难矛盾的能力。

（三）提升服务水平。进一步转变政府职能，更加重视履行社会管理和公共服务职责。推进新一轮政府机构改革，理顺部门职责关系，健全部门协调配合机制。深入开展机关作风评议活动，办好市县两级“行风热线”节目，推动“行风热线”进农村。完成市行政服务中心搬迁升级工作，抓好市直“一站式”电子政务二期工程，加快行政许可“绿色通道”建设，基本实现网上联合审批和网上行政审批在线监督。

（四）加强廉政建设。构建更加完善的惩治和预防腐败体系，严肃查处各类违法违纪案件。加强对落实“保增长”决策部署的监督，确保资金依法依规使用。整合纪检监察信访信息平台和农村党风廉政信息公开平台等监督资源，强化民生专项资金监管，深化惠农资金、涉农负担、教育收费、医疗卫生等方面的专项治理，坚决刹住损害群众利益的不正之风。

各位代表，我们要在中共肇庆市委的正确领导下，在市人大及其常委会、市政协的监督支持下，团结拼搏、开拓创新、真抓实干，全面完成今年经济社会发展目标任务，为把我市建设成为未来广东发展的新增长极和能够代表广东科学发展成果的城市作出新的贡献！
